# Acute-on-Chronic Liver Failure—Current Management and Future Perspectives

**DOI:** 10.3390/biomedicines13092193

**Published:** 2025-09-08

**Authors:** Benedict Allhoff, Christoph Neumann-Haefelin, Philipp Kasper

**Affiliations:** Department of Gastroenterology and Hepatology, Faculty of Medicine and University Hospital Cologne, University of Cologne, 50937 Cologne, Germany; benedict.allhoff@uk-koeln.de (B.A.); christoph.neumann-haefelin@uk-koeln.de (C.N.-H.)

**Keywords:** liver cirrhosis, complications, acute decompensation, liver failure, acute-on-chronic liver failure

## Abstract

Acute-on-chronic liver failure (ACLF) is a distinct clinical syndrome characterized by an acute decompensation of chronic liver disease in association with extrahepatic organ failure(s) and a high short-term mortality. Despite its increasing clinical relevance, there is no internationally standardized definition of ACLF to date. This review provides a comprehensive overview of current ACLF definitions, underlying pathogenic mechanisms, frequent precipitating events, and current treatment strategies. While liver transplantation remains the only curative treatment option, its role in the setting of ACLF is controversially debated, and patient selection remains complex due to high perioperative risk. Thus, the review article describes the current role of liver transplantation in patients with ACLF and describes novel prognostic scoring systems (e.g., TAM core, SALT-M model) that may be helpful in selecting suitable transplant candidates. Further emerging treatment options for ACLF include extracorporeal liver support systems, therapeutic plasma exchange, and immune-modulating approaches targeting toll-like receptor signaling that offer promising adjunctive strategies, though clinical evidence remains limited. Given the high burden and complexity of ACLF, harmonized definitions and evidence-based therapeutic frameworks are urgently needed to improve patient care and optimize transplant prioritization.

## 1. Introduction

The term acute-on-chronic liver failure (ACLF) describes a clinical syndrome characterized by an acute decompensation of a chronic advanced liver disease associated with organ failure(s) and high short-term mortality that was first mentioned by Ohnishi et al. in 1995 [1]. Since patients with ACLF have a poor prognosis, rapid identification of these patients is highly relevant in everyday clinical practice. Currently, there are various definitions of ACLF worldwide. The definitions currently most commonly used are presented in more detail below.

### 1.1. Definitions of ACLF

The definition proposed by the European Association for the Study of the Liver–Chronic Liver Failure (EASL–CLIF) Consortium originates from the prospective EASL-CLIF Consortium Acute-on-Chronic Liver Failure (CANONIC) study conducted in 2013 [2]. This large study, including 1343 patients with acutely decompensated cirrhosis across 29 liver units in eight European countries, aimed to identify diagnostic criteria for ACLF. To this end, the authors established a special scoring system, the chronic liver failure-sequential organ failure assessment (CLIF-SOFA) score, to diagnose ACLF, based on analyses of information from patients with organ failure and a high 28-day mortality rate (>15%), which now forms the basis of the EASL ACLF definition.

The CLIF-C organ failure (OF) scoring system includes information on six major organ systems (liver (bilirubin), kidney (creatinine), cerebral (grade of hepatic encephalopathy), coagulation (international normalized ratio), circulation (mean arterial pressure), and respiratory function (PaO_2_/FiO_2_, SpO_2_/FiO_2_)), whereby different cut-offs are used to define the presence of a specific organ failure [2]. The EASL definition of ACLF includes cirrhotic patients with and without prior decompensation [2]. The number of simultaneous organ failures, according to the CLIF-C OF score, is associated with an increasing 28-day mortality rate and stratifies the severity of the disease (ACLF Grade 1–3) [2]. While patients with ACLF grade 1 had a 28-day mortality rate of 22.1%, those with ACLF grade 3 exhibited a markedly higher 28-day mortality rate of 76.7% [2]. Table 1 provides an overview of the definition criteria and severity classification of ACLF according to the CLIF-OF organ failure score.

The EASL definition differs from other internationally used ACLF definitions, such as those from the North American Consortium for the Study of End-Stage Liver Disease (NACSELD) or Asian Pacific Association for the Study of the Liver (APASL), with heterogeneity in defining patients at risk for ACLF and varying cut-offs defining organ failure.

The NACSELD defines ACLF as the presence of two or more extrahepatic organ failures in patients with liver cirrhosis [3]. Looking at the origins of the NACSELD definition, the primary goal was to create a simplified, bedside-applicable model to identify patients with ACLF as early as possible [3]. To this end, prospectively collected data from 507 cirrhotic patients with infections (present at admission or acquired during hospitalization) were analyzed from the NACSELD database. Like the EASL definition, the NACSELD definition relies on the identification of organ failures in cirrhotic patients with acute decompensation. Organ systems considered within the NACSELD criteria include renal, respiratory, circulatory, and brain function [3]. Renal failure is defined by the requirement of renal replacement therapy (RRT), respiratory failure by the need for mechanical ventilation, cerebral failure by hepatic encephalopathy (HE) of grade 3 or 4 according to the West-Haven criteria, and circulatory failure as a mean arterial pressure below 60 mmHg or a reduction of at least 40 mmHg from baseline despite fluid resuscitation [3]. According to the NACSELD criteria, ACLF can be diagnosed when two or more organ failures are detectable [3].

Comparing the EASL and NACSELD ACLF definitions, they differ in the number of organ systems that can be affected (six (EASL) vs. four (NACSELD)) and regarding the assessment of organ dysfunction by using different definition criteria. A recent comprehensive analysis of more than 10,000 patient data sets from the United Network for Organ Sharing by Li et al. demonstrated that the EASL-CLIF criteria were more sensitive in detecting ACLF and showed superior predictive performance for both all-cause and short-term mortality when compared to the NACSELD criteria [4].

The APASL ACLF definition, first published in 2009, was initially based on an expert consensus rather than patient data, whereby later updates in 2014 and 2019 incorporated statistical validation from large cohorts of ACLF patients. While both the EASL and NACSELD definitions include cirrhotic patients regardless of prior decompensation, the APASL definition focuses on patients with compensated chronic liver disease (either cirrhotic or non-cirrhotic) who experience an acute hepatic decompensation due to a direct hepatic insult and associated liver dysfunction [5]. According to the APASL definition criteria, ACLF is diagnosed when jaundice (total bilirubin ≥ 5 mg/dL) and coagulopathy (INR ≥ 1.5 or prothrombin activity < 40%) are present, followed by the development of ascites, encephalopathy, or both within four weeks [5]. An overview of the different international ACLF definition criteria is shown in Table 2.

Unfortunately, to date, there is no internationally standardized definition of an ACLF, reflecting ongoing controversy in the field. While the American Association for the Study of Liver Diseases (AASLD) and EASL define ACLF with both intra- and extrahepatic perspectives in identifying precipitants and organ involvement, the APASL-ACLF definition is restricted to patients with acute liver dysfunction triggered by acute intrahepatic precipitants, with no prior decompensation episode. However, despite differences in the specific definition criteria between the individual definitions of ACLF, they also have key similarities. All definitions emphasize an acute onset, the presence of decompensated liver disease, and a high associated short-term mortality.

### 1.2. Concepts for a Harmonized ACLF Definition

Nonetheless, the harmonization of these definitions remains a longstanding objective, as a unified international standard definition would enhance the comparability of study outcomes, particularly in therapeutic research. Harmonizing the definition by reclassifying the existing definitions as an ACLF-Type A and Type B has been recently proposed by Kulkarni and Sarin [6]. The authors suggest that the existing three definitions should not be used to distinguish different entities but to describe different stages of the complex ACLF. Within the proposed harmonization model, ACLF type A corresponds to the APASL criteria and is graded using the APASL ACLF Research Consortium (AARC) score (grades A1–A3) [6]. This type excludes patients with prior hepatic decompensation or extrahepatic insults and is typically reversible. In ACLF type A, infections are primarily seen as complications rather than precipitants [6]. In contrast, ACLF type B aligns with the EASL-CLIF framework (grades B1–B3) based on concomitant organ failures, encompassing patients with prior decompensation and organ failures stemming from both hepatic and extrahepatic causes, including infections [6]. Thus, as per the harmonization model of Kulkarni and Sarin, patients with ACLF can progress from stage A to B as the disease progresses. However, this attempt to harmonize definitions remains a theoretical construct so far and needs to be further conceptualized and tested in future studies.

## 2. Epidemiology

Liver diseases, and, in particular, liver cirrhosis, are among the leading causes of death worldwide [7,8,9]. In the context of ACLF, the interpretation of epidemiological data is complicated due to the heterogeneity of existing definitions. A recent systematic review and meta-analysis, including 18,400 patient data sets, estimated that approximately 35% of hospitalized patients with decompensated cirrhosis meet the diagnostic criteria for ACLF as defined by the EASL-CLIF consortium [10]. Considering severity, ACLF grade 1 was the most frequently observed subtype globally, accounting for approximately 44% of cases, followed by grade 2 (32%) and grade 3 (21%) [10]. The distribution of ACLF grades varied considerably across different global regions. While grade 1 predominated in Europe and America, grade 2 was most prevalent in East Asia, and grade 3 was more common in South Asia [10].

In Europe, the prevalence of ACLF in decompensated cirrhosis was estimated at 39%, slightly exceeding the global average. Notably, this prevalence was only surpassed in South Asia, where a prevalence of 65% was reported [10]. Within Europe, the CANONIC study, which included data from Belgium, France, Germany, Italy, Spain, and the United Kingdom, revealed that Germany had the fourth-highest prevalence among the participating countries [2]. According to the German cohort within the CANONIC study, the reported prevalence of ACLF was 34.2% [2]. However, country-specific epidemiological data for Germany remain scarce and are generally derived from broader European studies. This limited availability of national data underscores the need for further regionally focused epidemiological research in Germany.

## 3. Pathogenesis

Hallmarks of ACLF pathogenesis include systemic inflammation, immune dysfunction, and increased susceptibility to infections. These factors, triggered by an acute insult in a patient with chronic liver disease, lead to hepatic injury, organ failure, and high mortality.

Typically, patients presenting with liver cirrhosis are already in a state of cirrhosis-associated immune dysfunction (CAID), which becomes worse with increasing liver dysfunction [11]. While about 1% of patients with compensated cirrhosis display signs of systemic inflammation, this number increases up to 50% in patients with decompensated cirrhosis [11,12]. As a sign of severe CAID, patients with decompensated cirrhosis and ACLF reveal elevated white blood cell counts and significantly increased serum C-reactive protein levels compared to patients with acute decompensation without ACLF [13]. An important pathogenic mechanism in the development of systemic inflammation associated with liver cirrhosis is the translocation of bacteria or pathogen-associated molecular patterns (PAMPs), such as lipopolysaccharides (LPS), from the intestine into the portal circulation [14]. Once bacteria or bacterial products, such as LPS, enter the portal bloodstream, PAMP-associated signaling pathways are activated. The subsequent sustained activation of the immune system exceeds its regulatory capacity, leading to an exaggerated and dysregulated immune response that is energetically demanding, ultimately resulting in mitochondrial dysfunction and multi-organ failure [11,15]. Moreover, the inflammatory milieu promotes necrotic cell death, which in turn releases damage-associated molecular patterns (DAMPs), which further increase systemic inflammation [16]. Both PAMPs and DAMPS are recognized by pathogen recognition receptors (PRRs) such as toll-like receptors (TLRs), such as TLR subtype 4 (TLR4). TLR4, located on the cell surface, serves as the key PRR detecting lipopolysaccharides [17]. TLR stimulation further amplifies inflammation by promoting the production of pro-inflammatory cytokines [17].

In summary, ACLF is characterized by profound immune dysregulation, including disturbances in both immune cell function and cytokine signaling. These alterations represent potential targets for therapeutic intervention. Understanding the pathophysiological mechanisms underlying ACLF is essential not only for the management of acute episodes but also for the development of future therapeutic approaches.

## 4. Precipitating Events

Effective management of patients presenting with ACLF starts with the identification of the underlying precipitating event. Understanding these precipitating triggers is crucial, as they play a central role in the clinical course of ACLF. The most common precipitating factors include (bacterial) infections, alcohol-related hepatitis, gastrointestinal hemorrhage, drug-induced liver injury, and an acute flare of chronic liver disease, such as hepatitis B virus infection or autoimmune hepatitis [18].

Recent studies have demonstrated marked geographical variations in the nature and frequency of these events [10]. In the Asian population, flares of chronic hepatitis B infection represent the most common precipitant [10]. In contrast, data from Western populations identified bacterial infections, alcohol-related hepatitis, gastrointestinal hemorrhage, hepatitis E virus infection, and drug-induced acute encephalopathy (e.g., due to sedatives) as the most common precipitating causes [18]. This is similar in the United States (US), where 41.6% of ACLF cases are precipitated by gastrointestinal bleeding, alcohol use, and infections [19]. Interestingly, despite intensive diagnostic efforts, no precipitating factor can be identified in around 30–50% of decompensated cirrhosis cases [2,18].

An interesting trend emerges when stratifying patients by ACLF severity, as the likelihood of identifying a precipitating event increases with disease severity. The proportion of cases with no identifiable trigger decreases from 51.4% in grade 1 ACLF to 40.0% in grade 2 and 27.3% in grade 3, suggesting a correlation between disease severity and the detectability of the underlying precipitating cause [2]. Nonetheless, the early identification of potential triggers remains essential for guiding clinical decision-making and tailoring therapeutic strategies. The key aspects of the most common precipitating events are briefly discussed below.

Bacterial infections emerge as the leading cause of ACLF development and are associated with a poor outcome [20]. The European CANONIC study initially reported a prevalence of 32.6%, a finding later corroborated by the PREDICT study, which observed bacterial infections in 44.1% of ACLF cases [2,18]. Notably, urinary tract infections were the most frequent infection source in pre-ACLF patients, followed by spontaneous bacterial peritonitis [21].

Alcohol-related hepatitis represents another major precipitant for ACLF. According to data from the PREDICT study, alcohol was responsible for 43.6% of ACLF episodes, underscoring its substantial role in disease progression [18].

Gastrointestinal bleeding associated with hypovolemic shock was identified as the third most frequent trigger of ACLF, accounting for 5.9% of cases [18].

Importantly, a considerable proportion of patients (24.8%) presented with two or more concomitant precipitants, further complicating clinical management [18].

While the majority of ACLF cases are driven by a defined set of common triggers, the current EASL clinical practical guidelines on ACLF also recommend evaluating less frequent etiologies when standard work-up fails to identify a precipitating event. These include intra- and extrahepatic viral infections (e.g., Epstein–Barr virus, cytomegalovirus, hepatitis A–E), Wilson’s disease, autoimmune hepatitis flares, and ischemic hepatitis [22]. Moreover, nutritional supplements should be recognized as potential precipitants of hepatic decompensation and thus considered as triggering factors of ACLF. Reports have documented that several hepatotoxic agents, such as ashwagandha or turmeric, can trigger decompensation in patients with liver cirrhosis [23].

In summary, the identification of precipitating events is a crucial step in the management of ACLF. Recognizing the regional epidemiology, prioritizing common causes, and remaining vigilant for rare triggers are important to optimize patient outcomes in this high-risk population.

## 5. Management

### 5.1. General Management

#### Intensive Care Monitoring

After the diagnosis of ACLF has been established, it is essential to evaluate the most appropriate clinical setting for the patient’s further management. Patients with ACLF should be considered for admission to an intermediate or intensive care unit (IMC, ICU), respectively, and ideally transferred to a transplant center. Key considerations regarding the setting of further monitoring include hemodynamic stability and the need for mechanical ventilation, underlying risk factors, existing comorbidities, and the severity of the underlying ACLF. Several scoring systems can assist in this assessment, such as the CLIF-C-OF score, the CLIF-C ACLF score, and the Model for End-Stage Liver Disease (MELD) score. However, as no definitive cut-off values for a transfer to an ICU or IMC unit exist, decisions regarding ICU admission must be made on a case-by-case basis. According to the EASL clinical practical guideline on ACLF, ICU admission is recommended within six hours of diagnosis when organ support is required [22]. Recommended indications for an immediate ICU admission include massive gastrointestinal bleeding, septic shock, and grade III–IV hepatic encephalopathy. Patients with previous but controlled variceal bleeding, grade II–III hepatic encephalopathy, sepsis without shock, hepatorenal syndrome with acute kidney injury (HRS-AKI), or liver failure (defined as bilirubin ≥ 12 mg/dL) without life-threatening complications can initially be monitored on an IMC unit [22]. A recently published meta-analysis of 13 studies confirmed that cirrhotic patients who are admitted to the ICU or IMC have a significantly better outcome than about 20 years ago, which particularly highlights the development in the field of intensive care medicine [24].

After patients with ACLF have been admitted to the ICU to receive specialized organ support, it is recommended to re-assess the ACLF severity and mortality risk regularly, since ACLF typically reveals a dynamic course [22]. Recent data from a multicenter trial of critically ill patients with decompensated cirrhosis in Europe and North America demonstrated that the 90-day mortality in patients with three or more organ failures (ACLF-3) was significantly lower in patients who showed improvement by day 3 compared with those who did not (40% vs. 79%) [25]. Therefore, a sequential reassessment and repetitive evaluation of prognosis (e.g., every 3–7 days) is warranted to adequately predict prognosis in these patients at risk. In patients with ACLF grade 3 and a CLIF-C ACLF score ≥ 70 who are not candidates for liver transplantation, the benefit of continuing intensive care management should be critically evaluated, and the initiation of palliative care should be considered [22].

### 5.2. Management of Precipitating Events and Complications

**Figure 1:**
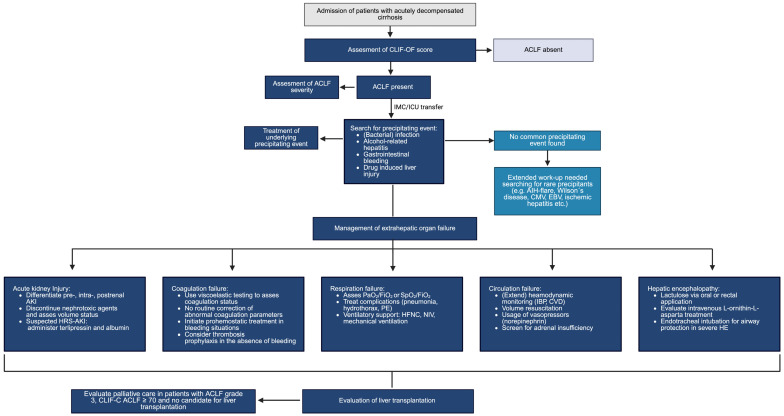
Management of ACLF.

Early identification of the precipitating event and accompanying complications is an essential aspect of management in patients with ACLF. The management of individual organ dysfunctions is addressed in more detail below, and Figure 1 presents a possible algorithm for the treatment of various organ dysfunctions in patients with ACLF.

#### 5.2.1. Bacterial Infections

As previously mentioned, infections, and particularly bacterial infections, represent the most common precipitating event for ACLF in Europe [2,18]. Therefore, a comprehensive diagnostic assessment of a patient with ACLF should always include the collection of blood cultures, urine, and ascites fluid samples for microbiological testing, in addition to a medical history and physical examination [22]. A chest X-ray is recommended as part of the initial diagnostic work-up, particularly in patients with respiratory symptoms [26]. If no infectious focus can be identified but clinical suspicion remains high, further diagnostic measures such as abdominal ultrasonography or computed tomography may be warranted. If a bacterial infection is suspected as the underlying precipitating event, initial therapeutic management should involve the administration of broad-spectrum antibiotics. The selection of an appropriate antibiotic regimen must take into account regional factors, local patterns of antimicrobial resistance, and patient-specific resistance profiles [22]. The critical importance of early broad-spectrum antibiotic initiation is underscored by a prospective study involving more than 1100 patients with decompensated cirrhosis, which demonstrated that extensively drug-resistant bacteria (XRD) were more frequently detected in patients with ACLF compared to those without ACLF [27].

#### 5.2.2. Alcohol-Associated Hepatitis

The second most common precipitating factor for ACLF is alcohol-associated hepatitis [18]. Therefore, an evaluation of recent alcohol consumption and the presence of alcohol use disorder should always be carried out in patients with ACLF. To detect alcohol consumption, an alcohol biomarker such as phosphatidylethanol or urinary ethyl glucuronide can be used [28]. According to recent guidelines for the management of alcoholic liver disease, glucocorticoid therapy is recommended for patients with severe alcohol-associated hepatitis with a Maddrey Discriminant Function score greater than 32 or a MELD score > 20, respectively, with reassessment of treatment response after four or seven days using the Lille score [29,30,31]. However, it should be considered that the response rate to glucocorticoid therapy decreases with increasing severity of ACLF. While approximately 52% of patients with ACLF grade 1 respond to steroid treatment, the response rate drops to only 8% in patients with ACLF grade 3 [32]. The incidence of infections associated with glucocorticoid treatment is estimated at around 33%, representing a major threat, especially for ACLF patients [33]. Therefore, glucocorticoids should be used with great caution in this setting, accompanied by close monitoring for infections. The EASL ACLF clinical practice guideline advises against the use of glucocorticoid therapy in patients with ACLF grade 3 due to a low response rate [22].

#### 5.2.3. Variceal Hemorrhage

Despite significant advances in both pharmacological and interventional treatment options over recent decades, gastrointestinal and portal hypertensive bleeding remains another major clinical challenge and is the third most common precipitating event in ACLF [18]. Endoscopic management is considered the first-line option for both upper and lower gastrointestinal bleeding to control bleeding [22]. However, transjugular intrahepatic portosystemic shunt (TIPS) placement also plays an important role, not only as a rescue therapy in cases of uncontrollable bleeding but also in reducing the risk of re-bleeding, which is approximately twice as high in patients with ACLF [34]. The Baveno VII consensus recommends pre-emptive TIPS placement in patients with a high risk of re-bleeding (Child–Pugh C score of less than 14 or a Child–Pugh B score greater than 7 with active bleeding at initial endoscopy or a hepatic venous pressure gradient (HVPG) exceeding 20 mmHg during hemorrhage) [35]. In patients with ACLF grades 1 and 2, multiple studies have demonstrated that TIPS placement is safe and significantly improves outcomes after variceal hemorrhage [34,36]. Therefore, the EASL ACLF guideline even recommends pre-emptive and rescue TIPS evaluation in ACLF patients with bilirubin levels exceeding 5 mg/dL (otherwise considered a contraindication for TIPS) [22]. However, it should be considered that none of these studies included patients with ACLF grade 3. Therefore, TIPS intervention in patients suffering from ACLF grade 3 should be considered very critical.

#### 5.2.4. Acute Kidney Injury

Acute kidney injury (AKI) is the most frequent extrahepatic organ failure in ACLF patients, with a prevalence ranging from 29% to 75%, defined by EASL-CLIF criteria [2,37,38,39]. Typically, the three main causes of AKI include renal hypoperfusion due to hypovolemia (pre-renal), structural kidney injury (intra-renal), and urinary obstruction (post-renal). In patients with cirrhosis and ascites, acute kidney injury (AKI) may also occur as hepatorenal syndrome (HRS), representing a specific form of AKI (HRS-AKI) that is associated with increased morbidity and mortality. HRS-AKI is characterized by an increase in serum creatinine ≥ 0.3 mg/dL within 48 h or ≥50% from baseline value within the prior 7 days and/or urine output ≤0.5 mL/kg/body weight for ≥6 h and no improvement in serum creatinine and/or urine output within 24–48 h following adequate volume resuscitation in patients with decompensated cirrhosis and ascites [40]. When HRS is diagnosed according to the criteria of the International Ascites Club, rapid initiation of therapy is urgently required, since acute kidney failure can adversely affect the prognosis of ACLF. The cornerstone of HRS management is a combination of (splanchnic) vasoconstrictor therapy and albumin administration [41]. The two most commonly used vasoconstrictors in HRS-AKI management include terlipressin (2–12 mg/day) and norepinephrine (0.5–3 mg/h) [42]. Even though terlipressin is the most commonly used pharmakon in the presence of HRS-AKI, it is important to note that terlipressin has been associated with increased colonization by multidrug-resistant bacteria and appears to be less effective with increasing ACLF severity [43,44]. When administering albumin (20–40 g/day), it must be considered that this therapeutic approach carries a risk of developing pulmonary edema, which may further impair respiratory function in already critically ill patients with ACLF. Further treatment options for HRS-AKI include renal replacement therapy if pharmacotherapy fails [45].

#### 5.2.5. Hepatic Encephalopathy

Brain failure is defined according to the EASL-CLIF criteria as grade 3 or 4 hepatic encephalopathy based on the West-Haven classification [2]. In cirrhotic patients presenting with altered mental status, HE should always be considered as an underlying cause. However, alternative causes such as alcohol intoxication, electrolyte imbalances, or intracranial hemorrhage must also be ruled out. In patients with suspected HE, a comprehensive diagnostic workup is essential and should include laboratory testing, a detailed medication and substance use history, and, if necessary, neuroimaging to exclude alternative causes of neurological impairment [45]. Although frequently performed in clinical practice, the measurement of ammonia levels is not recommended due to limited diagnostic and prognostic utility [46]. Once HE is identified as the underlying cause of an altered mental status in patients with ACLF, treatment should be initiated promptly, alongside the identification and management of precipitating factors. First-line therapy consists of lactulose via oral or rectal administration [46]. Additional therapies, such as L-ornithine L-aspartate (LoLa), have been proposed, though their clinical efficacy remains a subject of ongoing debate. A recent study analyzing the role of intravenous LoLa in cirrhotic patients with severe hepatic encephalopathy (grade III-IV) demonstrated that a combination of LoLa (30 g/24 h) with lactulose and rifaximin was more effective than only lactulose and rifaximin in improving grades of HE, recovery time from encephalopathy, and improved 28-day survival [47,48]. In cases of severe HE with a high risk of aspiration, endotracheal intubation for airway protection should be considered [45].

#### 5.2.6. Coagulation Failure

According to the EASL-CLIF criteria, coagulation failure is defined as an international normalized ratio (INR) of ≥2.5 [2]. However, it is important to note that the INR only reflects procoagulant factors, specifically factors I, II, V, VII, and X, while failing to account for anticoagulant proteins such as protein C and protein S. As a result, INR levels do not reliably correlate with the actual bleeding risk of cirrhotic patients with ACLF [49]. While conventional laboratory findings often suggest an increased bleeding risk, patients with liver cirrhosis, particularly those with ACLF, are also at increased risk of thrombotic complications [49]. This apparent paradox has prompted the exploration of more comprehensive coagulation assessment tools, such as rotational thromboelastometry (ROTEM) [50]. However, despite its potential to assess a hepatic coagulopathy more adequately, ROTEM has not yet been widely adopted in routine clinical practice. The role of individual coagulation factors in predicting the risk of ACFL remains unclear. Whereas factor V is well established as a prognostic marker in acute liver failure, comparable evidence supporting its role in ACLF is currently lacking and requires further investigation [51]. In general, coagulation correction should not be performed in the absence of active bleeding in patients with ACLF. Prophylactic anticoagulation should not be withheld, as it has been shown to reduce the risk of venous thromboembolism without significantly increasing the risk of bleeding [16,52].

#### 5.2.7. Respiratory Failure

Respiratory failure is another relevant complication in patients with ACLF, since these patients are prone to developing acute lung injury that can potentially progress to acute respiratory distress syndrome (ARDS) [53]. Recently, Schulz and co-workers showed that mechanical ventilation and pulmonary failure were identified as independent risk factors for increased short-term mortality in patients with ACLF [54]. According to the EASL-CLIF criteria, respiratory failure is defined as a PaO_2_/FiO_2_ ratio ≤ 200 or an SpO_2_/FiO_2_ ratio ≤ 214 [2]. In cirrhotic patients, and particularly in those with ACLF, evaluation of respiratory failure should include assessment for hepatopulmonary syndrome, porto-pulmonary hypertension, and hepatic hydrothorax, particularly if there is no alternative cause of respiratory failure such as pneumonia. Management strategies for respiratory failure involve both supportive and targeted therapies. In cases of hepatic hydrothorax or significant ascites, therapeutic paracentesis may improve pulmonary mechanics. Respiratory support ranges from non-invasive ventilation to high-flow nasal cannula (HFNC), with studies showing no significant difference in terms of intubation rates or mortality between the two modalities [55]. In patients with HE, HFNC is favored by the recent AASLD ACLF guideline due to a potentially lower risk of aspiration [45]. If invasive mechanical ventilation becomes necessary, standard recommendations from general critical care guidelines should be followed, including lung-protective ventilation.

#### 5.2.8. Circulatory Failure

Circulatory failure in ACLF is defined by the requirement for vasopressor therapy, according to the EASL-CLIF criteria [2]. Systemic inflammation as a hallmark of ACLF pathogenesis leads to reduced vascular resistance, arterial hypotension and increased cardiac workload and is therefore a key factor in developing circulatory failure [56]. In addition, adrenal insufficiency is frequently observed in critically ill patients with decompensated cirrhosis, aggravating circulatory dysfunction [57]. Accordingly, the current AASLD guidelines recommend routine screening for adrenal insufficiency in ACLF patients presenting with circulatory failure, as early diagnosis allows for timely and targeted treatment [45]. The general management of circulatory failure aligns with the standard critical care protocols, including norepinephrine as the first-line vasopressor and close monitoring of circulatory function.

### 5.3. Liver Transplantation

The only definitive curative treatment for ACLF remains liver transplantation. However, patients with ACLF represent a special risk group, as they have a high perioperative risk.

The long-standing assumption that patients with cirrhosis and concurrent organ failure are unsuitable transplant candidates has been refuted by multiple studies demonstrating one-year survival rates of 80% or higher in patients with ACLF who underwent liver transplantation [58,59,60,61]. Long-term follow-up studies further support these findings, showing three- and five-year survival rates reaching up to 70% [62,63]. This is particularly relevant considering that the majority of patients with ACLF are 60 years of age or younger [64]. However, a recent meta-analysis comparing outcomes in 22,238 patients with ACLF to 30,791 liver transplant recipients without ACLF found that post-transplant survival was significantly reduced in the ACLF group [65]. Specifically, one- and five-year survival rates were 86.0% and 66.9% in ACLF patients, compared to 91.9% and 80.7% in patients without ACLF [65]. Therefore, it is of utmost importance to identify those patients who are likely to achieve favorable post-transplant outcomes.

ACLF in the context of alcohol-associated liver cirrhosis represents a particular clinical challenge. In Germany, a six-month period of abstinence is generally required prior to transplant listing [66]. However, in patients with ACLF, such a delay may be fatal. The recent EASL clinical practice guidelines on liver transplantation propose several criteria that may justify an evaluation of liver transplantation despite prior alcohol use, including a lack of prior knowledge of liver disease, the absence of psychiatric comorbidities, and the presence of a supportive family network [67,68]. In this setting, the situation must be assessed on a case-by-case basis, and multidisciplinary counseling, including a thorough psychosocial assessment and close involvement of mental health care providers, is of paramount importance.

Most national or supranational organ allocation systems rely on the MELD or MELD-Na score. However, these scores do not adequately account for the manifestation of extrahepatic organ failures. This limitation of the current allocation scores becomes particularly evident in critically ill patients with higher ACLF grades. For instance, patients with ACLF grade 3 and MELD-Na scores between 20 and 29 experience a mortality rate of 64%, despite the MELD-Na score predicting a 3-month mortality of only 20% [69]. This discrepancy is increasingly recognized by national allocation systems, with the United Kingdom leading the way by incorporating ACLF as an additional factor in organ allocation criteria [70]. In line, the Spanish Society for Liver Transplantation proposed prioritizing patients with ACLF grade 3 when fit for surgery [70]. However, it is important to consider the opposing perspective: prioritizing ACLF patients may inadvertently disadvantage individuals who, in general, have a more favorable post-transplant prognosis.

When evaluating ACLF patients for liver transplantation, both risk and protective factors must be carefully weighed to predict post-transplant outcomes. Risk factors for an adverse outcome include older age (>60 years), diabetes, obesity, and concomitant cardiovascular diseases such as coronary heart disease and cardiac arrhythmias [59,71,72]. The patient’s general condition is also crucial. Frailty, sarcopenia, and low performance status are associated with reduced post-transplant survival [73,74]. According to international expert consensus, a Clinical Frailty Scale (CFS) score of ≥7, when assessed prior to ICU admission, should be considered a contraindication for liver transplantation in ACLF patients [75]. Other detrimental factors include portal vein thrombosis and respiratory compromise, particularly in cases requiring intubation or mechanical ventilation [72,76]. Conversely, several protective factors have been identified that suggest a favorable post-transplant prognosis in patients with ACLF. These include stabilization or even improvement of organ failure prior to transplant, low arterial lactate levels, and a short waiting time on the waitlist [59,72].

The role of donor characteristics remains controversial. Although some authors have proposed that ACLF recipients benefit from a donor risk index below a certain threshold, larger studies have failed to confirm this association [77,78].

Overall, defining the ideal ACLF liver transplant candidate among ACLF patients remains a major clinical challenge. To aid in this decision-making process, several working groups have developed prognostic scoring systems, including the Transplantation for ACLF grade 3 Model (TAM) score, the Sundaram ACLF Liver Transplantation Model (SALT-M) score, and the UCLA-FRS score.

The TAM score was specifically designed for patients with ACLF grade 3 [79]. It incorporates variables such as age, need for mechanical ventilation, arterial lactate concentration, and leukocyte count [79]. However, while the TAM score appears helpful in identifying the optimal transplant window, it falls short in predicting one-year post-transplant mortality [80,81].

To address this limitation, the SALT-M score was introduced. The SALT-M model has been developed to predict one-year mortality following transplantation in patients with ACLF grades 2 and 3 [82]. The score includes age over 50 years, use of one or more inotropic agents, presence of respiratory failure, diabetes mellitus, and body mass index [82]. Previous studies demonstrated that the SALT-M score outperformed other established models, such as the MELD score, in predicting one-year mortality in patients with ACLF grades 2 and 3 [83]. However, despite encouraging preliminary results, validation in larger, more diverse patient populations is required to confirm its reliability in real-world clinical settings.

To date, none of these scoring systems has been fully integrated into routine clinical practice. However, they may still provide valuable guidance in the evaluation process, particularly when used in combination rather than as standalone tools.

Although liver transplantation represents a highly effective treatment option for patients with ACLF, the selection criteria for critically ill patients with ACLF remain insufficiently defined and require further refinement.

## 6. Future Perspectives

As mentioned above, the only rescue option for ACLF remains liver transplantation. However, due to the global shortage of donor organs, considerable hope has been placed in the development and application of extracorporeal liver support systems (e.g., DIALIVE device, MARS, Prometheus). A range of devices has been engineered, particularly targeting the pathophysiologically relevant aspects of ACLF, including immune dysregulation and systemic inflammation.

In a recent multicenter, randomized controlled trial, the DIALIVE support system, a liver dialysis device that aims to exchange dysfunctional albumin and remove DAMPs and PAMPs from the circulation, was evaluated against standard of care in patients with ACLF [84]. The study demonstrated that treatment with the DIALIVE liver dialysis device significantly reduced inflammatory markers and led to a more rapid resolution of ACLF compared to the standard of care group [84]. However, there were no significant differences in 28-day mortality [84]. Nevertheless, like most studies in the field of liver dialysis, this trial was limited by a relatively small sample size. However, although preliminary evidence suggests potential benefits of such therapeutic approaches, no general recommendation for their routine clinical use has yet been established [22].

Another promising treatment option in patients with acute liver failure and ACLF is therapeutic plasma exchange (PE). PE uses the principle of apheresis with removing toxins and proinflammatory factors, such as cytokines, and replacement with essential proteins using a combination of fresh frozen plasma (FFP) and 5% albumin solution [85,86]. In a recent meta-analysis of 20 studies including 5705 ACLF patients, PE was associated with a significantly higher 30-day and 90-day survival rate compared to standard medical therapy [87].

Stem cell therapy represents another promising option in the treatment of ACLF. Stem cells exhibit immunomodulatory and reparative properties, and their effects have also been explored in the setting of ACLF. In a meta-analysis, Xue et al. reported that stem cell therapy may confer short-term benefits in patients with ACLF by improving liver function and alleviating liver damage [88]. However, further clinical and preclinical studies are required to determine its therapeutic value.

Further emerging therapeutic approaches for ACLF focus on targeted interventions, particularly those modulating systemic inflammatory pathways involved in disease progression. As discussed before, toll-like receptor 4 (TLR-4) plays a pivotal role in orchestrating systemic inflammation [17]. The administration of the TLR-4 inhibitor TAK-242 alone or in combination with granulocyte-colony stimulation factor (G-CSF) has demonstrated promising effects in preclinical studies, notably reducing organ failure severity and improving outcomes in rodent models of ACLF, thereby representing a potential therapeutic candidate for future clinical application [17,89].

Looking ahead, future therapeutic interventions are likely to be conceptualized as bridge-to-transplant strategies, given the current lack of curative alternatives to actual liver transplantation. Further research, particularly large-scale clinical trials, is essential to determine whether these liver support systems can serve as viable curative alternatives or adjuncts to liver transplantation.

## Figures and Tables

**Table 1:** CLIF-C organ failure score and ACLF severity grading according to EASL guidelines.

CLIF-C Organ Failure Score				
Organ System	Variable	Scale		
		1 Point	2 Points	3 Points
Liver	Bilirubin (mg/dL)	<6.0	≥6.0 to <12.0	≥12.0
Kidney	Creatinine (mg/dL)	<1.5	≥2.0 to <3.5	≥3.5 or use of RRT
		>1.5 to <2.0		
Coagulation	INR	<2.0	≥2.0 to <2.5	≥2.5
Cerebral	HE grade (West-Haven criteria)	0	I–II	III-IV or intubation for HE
Respiration	PaO_2_/FiO_2_ SpO_2_/FiO_2_	>300 >357	>200 to ≤300 >214 to ≤357	≤200 ≤214 Or use of mechanical ventilation
Circulation	Mean arterial pressure (mmHg)	≥70	<70	Use of vasopressors
**ACLF severity**				
ACLF 1a	Kidney injury (creatinine ≥ 2.0 mg/dL)			
ACLF 1b	1 organ failure + creatinine (>1.5 mg/dL to <2.0 mg/dL) or HE I-II			
ACLF 2	2 organ failures			
ACLF 3a	3 organ failures			
ACLF 3b	≥4 organ failures			

Abbreviations: FiO_2_, fraction of inspired oxygen; HE, hepatic encephalopathy; INR, international normalized ratio; PaO_2_, partial pressure of arterial oxygen; RRT, renal replacement therapy; SpO_2_, peripheral capillary oxygen saturation.

**Table 2:** Comparison of international definitions.

	EASL		NACSELD		APASL	
Foundation	CANONIC study		NACSELD database		Initially expert consensus, later statistical validation	
Stage of liver disease	Cirrhosis		Cirrhosis		Chronic liver disease	
Decompensation in patient’s history	With or without		With or without		No prior decompensations	
Criteria for ACLF	Minimum 1 OF		Minimum 2 OF		Liver failure and coagulation failure with the development of Ascites, HE or both within 4 weeks	
Organ failures	Liver	TB ≥ 12 mg/dL	Liver	N/A	Liver	TB ≥ 5 mg/dL
	Kidney	Crea ≥ 2 mg/dL or RRT	Kidney	RRT	Kidney	N/A
	Brain	WH III/IV	Brain	WH III/IV	Brain	Encephalopathy
	Coagulation	INR ≥ 1.5	Coagulation	N/A	Coagulation	INR ≥1.5
	Circulation	Usage of inotropes	Circulation	Usage of inotropes	Circulation	N/A
	Respiration	Mechanical ventilation or PaO_2_/FiO_2_ <200	Respiration	Mechanical Ventilation	Respiration	N/A
Prognosis	28-day mortality Grade 1: 22% Grade 2: 32% Grade 3: 77%		30-day mortality Grade 1: 49% Grade 2: 64% Grade 3: 77%		30-day mortality Grade 1: 13% Grade 2: 45% Grade 3: 86%	

Abbreviations: APASL, Asian Pacific Association for the Study of the Liver; CANONIC, Consortium of Acute-on-Chronic Liver Failure in Cirrhosis; FiO_2_, fraction of inspired oxygen; NACSELD, North American Consortium for the Study of End-Stage Liver Disease; PaO_2_, Partial Pressure of Arterial Oxygen; N/A, not applicable; WH, West-Haven; TB, Total Bilirubin; RRT, Renal Replacement Therapy; INR, International Normalized Ratio; HE, Hepatic Encephalopathy; OF, Organ failure.

## Data Availability

As this is a review article, no data were created.
